# Exposure to emissions from Mount Etna (Sicily, Italy) and incidence of thyroid cancer: a geographic analysis

**DOI:** 10.1038/s41598-020-77027-9

**Published:** 2020-12-04

**Authors:** Paolo Boffetta, Lorenzo Memeo, Dario Giuffrida, Margherita Ferrante, Salvatore Sciacca

**Affiliations:** 1 Stony Brook Cancer Center and Department of Family, Population and Preventive Medicine, Stony Brook University, Lauterbur Dr., Stony Brook, NY 11794 USA; 2 Department of Medical and Surgical Sciences, University of Bologna, Bologna, Italy; 3 Department of Experimental Oncology, Istituto Oncologico del Mediterraneo, Viagrande, Italy; 4 Department of Medical, Surgical Sciences and Advanced Technologies “G.F. Ingrassia”, University of Catania, Catania, Italy

**Keywords:** Oncology, Risk factors

## Abstract

An increased incidence of thyroid cancer has been reported in the area close to Mount Etna, the largest volcano in Europe located in Northeastern Sicily. We tested the hypothesis that exposure to the emissions from the volcano is associated with thyroid cancer in 186 municipalities from three provinces surrounding the volcano (1.9 million inhabitants). We measured the angle between the bearing of the municipalities and each direction, with special focus on South-East, the prevalent direction of the plume, and conducted a regression analysis on 2003–2016 incidence rates of thyroid cancer, adjusting for distance from Mount Etna, population size, and income. A 10-degree increase in the angle with South-East was associated with a decrease in thyroid cancer rates in the whole population (− 0.67 cases/100,000, p = 0.002) and in women (− 1.54/100,000, p < 0.001), and were robust to several sensitivity analyses. Similar results were obtained for East-South-East direction. These results support the hypothesis of a potential role of exposure to the plume from Mount Etna in determining the high rates of thyroid cancer. The results need to be confirmed in analytical studies, in which information of exposure to chemicals originating from the volcano, as well as other possible causes, should be carefully measured, molecular characteristics of the tumors and taken into account.

## Introduction

Mount Etna is the largest active volcano in Europe, located in the province of Catania in Northeastern Sicily. Gas emissions from its active summit craters contain a sizable amount of heavy metals and halogens, including iodine^1,2^, resulting in measurable human exposure (e.g.^3^). Several studies have estimated the proportion that is deposited on the ground in various forms (e.g., aerosol, solid crystals)^4^, and the resulting contamination of ground water^5^. Previous studies have addressed the potential impact of Etna volcanic emissions on human health, including multiple sclerosis^6,7^, amyotrophic lateral sclerosis^8^, and several cancers^9^, including thyroid cancer^10–13^. In particular, an analysis of 2002–2004 incidence of thyroid cancer in Sicily identified a higher rate in Catania province, which was attributed to papillary cancer and was not explained by mild iodine deficiency or industrial activities^10^. These and other authors^11–13^ hypothesized a role of the volcanic environment, possibly related the presence of heavy metals and other carcinogens in the aquifer used for drinking water in the surrounding area. We aimed at testing the hypothesis that exposure to the emissions from Mount Etna is an additional route of exposure to carcinogens of volcanic origin that can explain the increased incidence of thyroid cancer, by analyzing the correlation between the incidence of the disease and the angle between the bearing of the municipalities and each direction, with special focus on South-East, the prevalent direction of the plume.

## Results

The incidence rate of thyroid cancer in the study population was higher than those reported in other areas of Sicily, and comparable to other high-incidence areas in other Italian regions (Table 1). The median number of annual cases of thyroid cancer by municipality was 0.71, and four municipalities had no cases of thyroid cancer during the study period. The median number of annual cases by health district was 12.82.

**Table 1:** Selected characteristics of the provinces included in the analysis.

Province	Inhabitants (2018)	N municipalities	N districts	Median distance between municipalities and Mount Etna (km)	Median angle between bearing of municipalities and SE	Average income (2010 €)	ASR thyroid cancer/100,000
Catania	1,103,917	58	9	21.2	46°	8893	24.0
Enna	164,788	20	4	52.0	116°	8231	17.6
Messina	622,962	108	8	42.7	119°	9935	19.8

*ASR* age-standardized rate, *SE* South-East direction.

**Figure 1:**
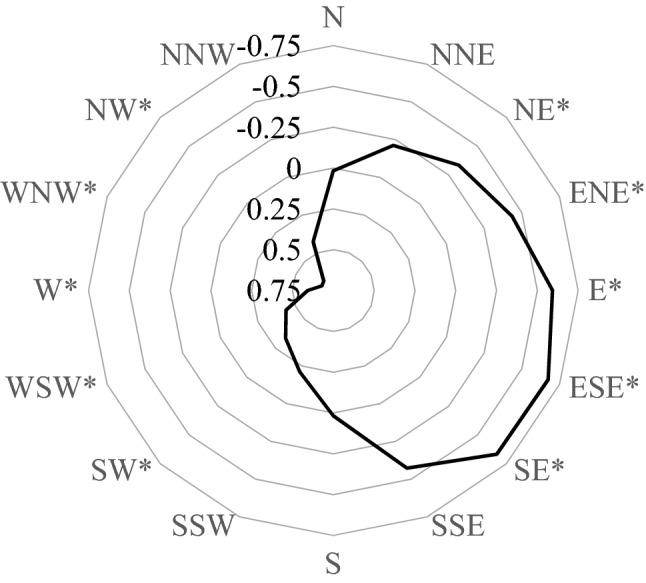
Radar chart of correlation coefficients between angle between bearing of municipality and cardinal directions and incidence of thyroid cancer (both sexes). * FDR-adjusted, p < 0.05.

The angles between bearing of the municipality of residence with respect to Mount Etna and the directions from between East-North-East and East-South-East were negatively associated with the incidence of thyroid cancer (Fig. 1); and the directions East-South-East and South-East were those with the strongest association (decrease of 0.7 cases/100,000 for a 10 degree increase in the angle). Detailed results for the South-East direction are reported in Table 2. In this analysis, a negative association was also detected between distance from Mount Etna and thyroid cancer incidence (decrease of one case/100,000 for a 10-km increase), although it was statistically significant only in men. Results for the East-South-East direction were very similar to those reported in Table 2.

**Table 2:** Results of regression analysis on incidence of thyroid cancer (South-East direction).

Level of analysis	Distance (10 km)		Angle between bearing and South-East (10°)	
	Coefficient	P-value	Coefficient	p-value
**Both sexes**				
Municipality	− 0.996	0.03	− 0.667	0.002
District	− 0.406	0.24	− 0.709	< 0.001
**Women only**				
Municipality	− 1.464	0.08	− 1.542	< 0.001
District	− 0.717	0.14	− 1.062	< 0.001

Similar results were obtained in the analysis by health district in both sexes combined and in women only. Results in men were excluded from the analysis because thyroid cancer rate in men were lower than those in women: in the province of Catania, the largest unit in our study, the rate during the study period was 11.1/100,000 in men and 36.1/100,000 in women. The sensitivity analyses, which was conducted on the South-East direction, provided results similar to those of the main analysis (Supplementary Table S1). When distance from Mount Etna and angle between bearing and South-East were included separately in the regression model, the p-value of the former decreased (correlation coefficient − 0.978, p = 0.01), while that of the latter did not (correlation coefficient − 0.704, p < 0.001), suggesting that results on distance from Mount Etna are partially confounded by the angle between bearing and South-East.

## Discussion

Our results show a decrease in the incidence of thyroid cancer of 0.67 cases/100,000 for a 10 degree increase in the angle between bearing of the place of residence from Mount Etna and either East-South-East or South-East, and of 1.00 case/100,000 for a 10-km increase in the distance from Mount Etna. Overall, these results support the hypothesis of a potential role of exposure to the plume from Mount Etna in determining the high rates of thyroid cancer observed in earlier analyses^9–14^. Both the distance from the volcano and the angle from the predominant direction of the plume were negatively associated with thyroid cancer rates, and the association was stronger for the latter.

We used municipality of residence as the primary unit of analysis. This approach allowed a detailed definition of both distance and angle, although it suffered from random fluctuation due the small size of some municipalities, leading to small number of cases of thyroid cancer. The analysis by health district, although based on a less detailed geographic assessment, was based on more stable numbers: the two sets of results were very similar in both sexes combined and in women.

Since incidence rates of thyroid cancer are higher in women are higher than in men, it is not surprising that the results restricted to women confirmed those of the analysis based on both sexes.

The hypothesis of a role of exposure to the plume from Mount Etna in determining the risk of thyroid cancer in the surrounding area is based on the detection of iodine^2,15^, heavy metals^1^ and radioactive compounds^16^ in the plume of this and other volcanoes. Higher levels of urinary cadmium, mercury, manganese, palladium, thallium, uranium, vanadium, and tungsten were detected in subjects leaving near Mount Etna compared to other areas of Sicily^9,12^. Two other routes of exposure to carcinogens originating from Mount Etna have been studies in the area: groundwater contamination, in particular by trace elements^5,10,17,18^, and soil gas emission of radon, as most of the faults are located on the Southeastern flank of the volcano^19^. Although volcanic elements can be transported by prevailing winds for several hundred kilometers, most of the deposition occurs within 100 km^20,21^.

Our study suffers from several limitations. We relied on an ecologic definition of both exposure and outcome. We classified individual subjects’ exposure based on the location of the city hall of the municipality of residence: not only study subjects, and in particular patients with thyroid cancer, could live away from the city hall (the average surface of the municipalities included in the study is 50.6 km^2^), but they can spend time outside the municipality of residence. Furthermore, the South-East direction is the prevalent but not the only one for crater plume dispersion^20,22^. Our results might have been affected by residual confounding. The incidence of thyroid cancer is higher in more affluent individuals^23^ and in urban areas. We attempted to control for these potential sources of confounding by adjusting for average income and population size. This inclusion of the terms for these two factors did not have an appreciable effect on the main variables of interest (distance from Mount Etna and angle between bearing and South-East direction), but adjustment at the ecologic level might not remove completely the effect of a confounder^24^. Exposure to ionizing radiation from medical, occupational or environmental sources, a known environmental cause of thyroid cancer, and iodine deficiency, a suspected cause of thyroid cancer, were not addressed in our analysis. In studies of thyroid cancer risk from environmental exposure, such as those conducted among atomic bomb survivors and in the Chernobyl population, the risk of thyroid cancer depended on age at exposure^25,26^. Future analytical studies in the population residing near Mount Etna should take this aspect into account, e.g., by separating those born and raised in the area from those who immigrated during adult life.

Papillary carcinoma of the thyroid is characterized by a high prevalence of *RET* chromosomal rearrangements, and of point mutations of *RAS* or *BRAF* proto-oncogenes, all of which are able to trigger the activation of mitogen-activated protein kinase (MAPK) cascade^27–29^, as well as *NTRK* fusions^30^. It is unknown whether tumors occurring in the area surrounding Mount Etna have different molecular characteristics compared to tumor from other areas.

In conclusion, our results support the hypothesis that exposure to the plume from Mount Etna, measured using both the angle from bearing of the municipality of residence from the volcano and the South-East direction, and the distance of municipalities from the volcano, is associated with increased incidence of thyroid cancer in the population of the three provinces surrounding it. Other sources of exposure to potential carcinogens originating either from Mount Etna—e.g., from drinking water^10,17^, soil^19^, or other sources such as type of employment, were not addressed in our analysis and remain valid hypotheses. To properly address the determinants of high incidence of thyroid cancer in the region of Mount Etna, analytical studies are needed, in which information on exposure to chemicals originating from the volcano, as well as other possible causes, including over-diagnosis^31,32^, should be carefully measured. In addition, molecular pathology might inform on whether tumors in the area have different characteristics, which may indicate a unique carcinogenic mechanism.

## Methods

We selected the population of three provinces of Catania, Enna and Messina in Northeast Sicily, surrounding Mount Etna, with a total population close to 1.9 million. We obtained from the population-based cancer registry of Eastern Sicily^33^ the number of cases and the age-standardized rates (European population standard) of thyroid cancer in each municipality and each health district of the three provinces for 2003–2016, for both sexes combined and for women only, as the small number of cases hampered the analysis restricted to men. We did not distinguish between histological types of thyroid cancer; however, most cases are papillary cancers^34^. Municipalities represent the basic administrative units in Italy, health districts are intermediate units which coordinate health care delivery within the national health system. Selected characteristics of the study area are reported in Table 3: the three provinces comprised 186 municipalities (median population, 3900) and 21 health districts (median population, 66,800).

**Table 3:** Incidence rate of thyroid cancer among women in selected Italian cancer registries, 2008–2012^6^.

Registry	ASR
Turin	18.8
Brescia	30.2
Milan	14.5
Veneto	20.7
Genoa	22.3
Parma	43.7
Romagna	42.3
Florence-Prato	27.5
Latina	42.0
Naples	21.2
Catania-Messina-Enna*	38.3
Palermo*	24.4
Ragusa-Caltanissetta*	22.7
Siracusa*	24.6
Nuoro	53.1
Average of 28 registries	28.2

*ASR* age-standardized rate/100,000.

*Cancer registries located in Sicily.

We calculated the distance between each municipality or district administrative center and Mount Etna, as well as the angle between their bearing from Mount Etna and each cardinal direction, with special focus on South-East (135°), the prevailing direction of plume of Mount Etna (Supplementary Fig. S1). We fitted generalized linear models to the municipal rates of thyroid cancer, including their standard errors as weights and using the *GLM* software package in Stata version 16^35^. In addition to distance and angle, we included in the regression models province, size of the population and mean per-capita income in 2010 (https://www.comuni-italiani.it/19/statistiche/). We intended to use the ‘deprivation index’^36^, but we were denied access to these data by the Epidemiology Service of Sicily Region. We chose this analytic approach because it provides results that are easily interpretable, and minimizes the assumptions. We applied the False Discovery Rate adjustment^37^ to the p-values of the analysis including all cardinal directions.

We conducted a secondary analysis, restricted to the South-East direction, based on health districts rather than municipalities, and four sensitivity analyses: (i) excluding the two largest municipalities, Catania (313,396 inhabitants) and Messina (236,962 inhabitants), (ii) restricting the analysis to the province of Catania, whose municipalities have the shortest distance from Mount Etna and the smallest angle from South-East (Table 3), (iii) excluding weights, and (iv) without adjustment for population size and income.

## Supplementary information

Supplementary Information 1.

Supplementary Information 2.

## Data Availability

All data used in the analysis are available without restrictions from the authors.
