# Supplementary material for: Exposure to emissions from Mount Etna (Sicily, Italy) and incidence of thyroid cancer: a geographic analysis
Source: Sci Rep. 2020 Dec 4;10:21298. doi: 10.1038/s41598-020-77027-9 (PMC7718918; doi:10.1038/s41598-020-77027-9)
Supplement: Supplementary file 1 — Supplementary Information 1. [file 41598_2020_77027_MOESM1_ESM.docx]

Supplementary Figure 1. Map of Sicily with example of distance from Mount Etna and angle from the South-East direction. The orange star indicates mount Etna and the red star the town of Nicolosi. Modified from Google Maps (Map data 2020 Google)
