# Supplementary material for: Exposure to emissions from Mount Etna (Sicily, Italy) and incidence of thyroid cancer: a geographic analysis
Source: Sci Rep. 2020 Dec 4;10:21298. doi: 10.1038/s41598-020-77027-9 (PMC7718918; doi:10.1038/s41598-020-77027-9)
Supplement: Supplementary file 2 — Supplementary Information 2. [file 41598_2020_77027_MOESM2_ESM.docx]

Exposure to emissions from Mount Etna (Sicily, Italy) and incidence of thyroid cancer: a geographic analysis

Paolo Boffetta [1, 2], Lorenzo Memeo [3], Dario Giuffrida [3], Margherita Ferrante [4], Salvatore Sciacca [3]

Supplementary Material

Supplementary Table 1. Results of sensitivity analyses, based on municipality of residence

| Level of analysis | Distance (10 Km) | | Angle between bearing and South-East (10°) | |
| --- | --- | --- | --- | --- |
|  | Coefficient | P-value | Coefficient | p-value |
| Excluding Catania and Messina |  |  |  |  |
| Both sexes | -0.725 | 0.06 | -0.652 | <0.001 |
| Women | -0.796 | 0.24 | -1.394 | <0.001 |
| Men | -0.623 | 0.10 | 0.111 | 0.53 |
| Only Catania province |  |  |  |  |
| Both sexes | -0.982 | 0.06 | -0.928 | 0.004 |
| Women | -1.883 | 0.01 | -1.329 | 0.003 |
| Men | -0.162 | 0.74 | -0.588 | 0.07 |
| Excluding weights |  |  |  |  |
| Both sexes | -0.727 | 0.06 | -0.644 | <0.001 |
| Women | -0.788 | 0.24 | -1.368 | <0.001 |
| Men | -0.624 | 0.10 | -0.105 | 0.55 |
| Not adjusted for income and population size |  |  |  |  |
| Both sexes | -0.789 | 0.04 | -0.697 | <0.001 |
| Women | -0.873 | 0.18 | -1.414 | <0.001 |
| Men | -0.663 | 0.08 | 0.058 | 0.73 |

Supplementary Figure 1.


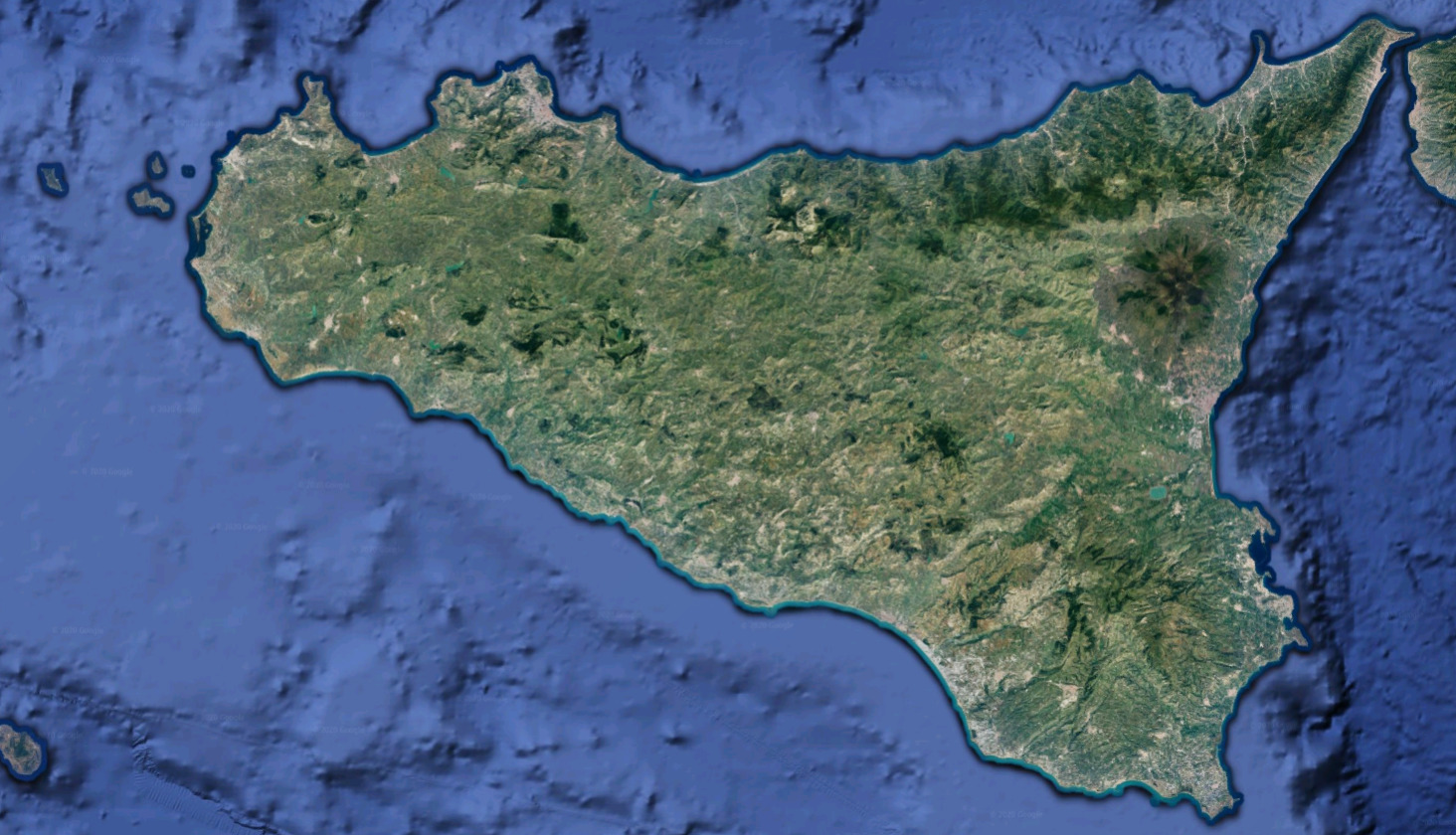


Nicolosi: bearing from Mount Etna: +166.3⁰

angle from SE: 31.3⁰

distance from Mount Etna: 15.1 Km

N

Nicolosi

Mount Etna

SE (+135⁰)
